# Human-Mimetic Estimation of Food Volume from a Single-View RGB Image Using an AI System

**DOI:** 10.3390/electronics10131556

**Published:** 2021-06-28

**Authors:** Zhengeng Yang, Hongshan Yu, Shunxin Cao, Qi Xu, Ding Yuan, Hong Zhang, Wenyan Jia, Zhi-Hong Mao, Mingui Sun

**Affiliations:** 1 College of Electrical and Information Engineering, Hunan University, Changsha 410082, China; 2 Department of Neurosurgery, University of Pittsburgh, Pittsburgh, PA 15260, USA; 3 Department of Electrical and Computer Engineering, University of Pittsburgh, Pittsburgh, PA 15260, USA; 4 School of Artificial Intelligence and Automation, Huazhong University of Science and Technology, Wuhan 430074, China; 5 Image Processing Center, Beihang University, Beijing 100191, China; 6 Department of Bioengineering, University of Pittsburgh, Pittsburgh, PA 15260, USA

**Keywords:** artificial intelligence, nutrition, food volume estimation, deep learning, dietary assessment

## Abstract

It is well known that many chronic diseases are associated with unhealthy diet. Although improving diet is critical, adopting a healthy diet is difficult despite its benefits being well understood. Technology is needed to allow an assessment of dietary intake accurately and easily in real-world settings so that effective intervention to manage being overweight, obesity, and related chronic diseases can be developed. In recent years, new wearable imaging and computational technologies have emerged. These technologies are capable of performing objective and passive dietary assessments with a much simplified procedure than traditional questionnaires. However, a critical task is required to estimate the portion size (in this case, the food volume) from a digital image. Currently, this task is very challenging because the volumetric information in the two-dimensional images is incomplete, and the estimation involves a great deal of imagination, beyond the capacity of the traditional image processing algorithms. In this work, we present a novel Artificial Intelligent (AI) system to mimic the thinking of dietitians who use a set of common objects as gauges (e.g., a teaspoon, a golf ball, a cup, and so on) to estimate the portion size. Specifically, our human-mimetic system “mentally” gauges the volume of food using a set of internal reference volumes that have been learned previously. At the output, our system produces a vector of probabilities of the food with respect to the internal reference volumes. The estimation is then completed by an “intelligent guess”, implemented by an inner product between the probability vector and the reference volume vector. Our experiments using both virtual and real food datasets have shown accurate volume estimation results.

## 1. Introduction

As of 2016, 39.6% U.S. adults were obese (BMI ≥ 30) [1]. In order to control obesity and related chronic diseases, there is a pressing need to assess accurately the energy and nutrient intake of individuals in their daily lives. Traditionally, a dietary assessment is conducted using self-report in which individuals report their consumed foods and portion sizes. Although this method is standard and has been utilized for decades, numerous studies have indicated that it is inaccurate and biased [2,3]. In addition, self-report does not work well in children [4].

With the development of smartphones and wearable devices, dietary assessment can be performed without fully depending on individuals’ memory and willingness to report their own intake. For example, Arab et al. [5] developed an automated image capture method to aid dietary recall using a mobile phone; Sun et al. [6] designed a wearable camera system called eButton for objective and passive dietary assessment; Jobarteh et al. [7] developed an eyeglass attachment containing an accelerometer and a camera to record dietary events automatically; and Liu et al. [8] performed food intake monitoring using a sensor worn on top of an ear.

With images automatically captured by a wearable device, dietary assessment can be conducted objectively and passively in four steps: Food detection, food recognition, volume estimation, and nutrition content analysis. The first two steps have been studied using computer vision and pattern recognition methods, notably the recently developed deep learning methods [9-11]. The last step is usually implemented using an existing food database, such as the USDA Food Composition Database [12]. Although the technological tools in all four steps need further improvements, the third step (volume estimation) is currently the least developed due to a number of challenges involved, which are detailed below.

A food image is usually in the unit of a “pixel”, rather than a real-world unit (e.g., “centimeter”). As a result, a scale reference is required to determine the actual food size. For example, the size of an apple must be determined by comparing it with another object with a known size in the same image. Thus, many types of fiducial markers have been used as the scale reference, such as a checkerboard [13] and a credit card like reference [3]. However, these fiducial markers must be carried by the individual and placed near the food before the individual starts eating, which is an unwelcome procedure usually difficult to implement. Secondly, to estimate food volumes effectively with a computer, it is necessary to provide enough three-dimensional (3D) information of the food. Unfortunately, much of the 3D information is lost in the imaging process where the food as a 3D object is projected to a 2D plane. For this reason, instead of using a single-view image, many researchers turned to the use of multiple images in different views [3,13-16]. However, this approach requires the individual to move either the camera or the food in the imaging process, complicating the research effort and possibly modifying the normal behavior of diet intake. In addition, 3D reconstruction from multi-view images involves multiple challenges, such as accurate camera calibration, feature extraction, image registration, pose estimation, etc. [17].

In another approach, a depth camera or a pair of cameras has been used to obtain images with depth information [18]. Although a depth image contains more 3D information, to acquire this type of images, the wearable device must be made larger, heavier, and more expensive to accommodate additional hardware and meet the requirement of increased power consumption, which affects the wearability and usability of the device.

With the success of deep learning-based depth estimation [19,20], it has been demonstrated that certain 3D information can be inferred from a 2D single-view image. Thus, there is a recent trend of applying deep learning to food volume estimation using estimated depth from the RGB image (i.e., the regular image with red (R), green (G), and blue (B) as three primary colors). Typical algorithms include im2calories [21] and deepvol [22]. Although these algorithms have achieved a certain success in improving food volume estimation, estimating depth from a single RGB image is still a very challenging problem. In addition, the deep learning system requires an excessively large number of RGB images with a known depth for network training, which are difficult to obtain in practice.

In this work, we present a human-mimetic approach to estimate food volume directly from a single-view 2D RGB image without using any supplemental 3D information. Our work is highlighted as follows.

1. Over the years, dietitians have used a popular portion size estimation method by comparing the observed food with a set of common objects of known volumes. We propose to use the AI technology to mimic this mental process. In the human case, the food size is first matched with the most similar size of a known object. In an AI system, we use a similar strategy formulated as an image classification step. In the second step, while the human mentally fine-tunes the estimation by portioning with respect to the known object sizes, we mimic this process using an inner product between a probability vector and a reference volume vector.
2. To validate the effectiveness of our method quickly, we used two large-scale Virtual Food Datasets (VFD), constructed by computer simulation, of different volume ranges. Our method achieves a high accuracy with an average volumetric error less than 9% on both datasets.
3. To evaluate the real-world performance of our method, two Real Food Datasets (RFD) are collected with different degrees of difficulties in estimation tasks. Our method achieves 11.6% and 20.1% mean relative volumetric errors on the easy and hard food datasets, respectively.

The rest of this paper is organized as follow. Food volume estimation methods related to our study are reviewed in Section 2. Section 3 describes the concepts of our volume estimation method. Constructions of VFD and RFD are detailed in Section 4. Experimental studies which validate our method are described in Section 5. Discussions of several important issues are provided in Section 6. Finally, conclusion and future directions are presented in Section 7.

## 2. Related Work

There have been considerable efforts on the application of the computer vision technology to food volume estimation. Existing methods can be roughly divided into two categories: Model-based methods and stereo vision-based methods. For the mode-based methods, the volume is estimated by registering each food in the input image with a pre-defined 3D food model. For example, Chen et al. [2] proposed a 3D/2D model-to-image registration to interactively match the food contour and the 2D projection of a 3D geometric model manually selected from a set of computer-generated shapes (a sphere, a wedge, a portion of spheroid, etc.). Limited by the prescribed set of 3D shapes, this method produces a large error when the shape of the food is highly irregular. Xu et al. [23] provided a solution by learning 3D food models from multi-view food images. Although improvements have been made, the model-based methods suffer from a common problem that an appropriate 3D model must be selected manually, which affects the data processing speed and is not cost-efficient. In order to solve this problem, stereo vision-based methods [3,13-16] have been developed to directly reconstruct the 3D food surface from a set of RGB or RGBD (a type of color image that contains the information of depth (D) at each pixel location) images. For example, Puri et al. [14] developed a reconstruction method from multiple images of different views. Similarly, Hassannejad et al. [13] generated a point cloud of food using six continuous frames extracted from a short video. To speed up the process of 3D reconstruction, Dehais et al. [3] proposed a two-view 3D reconstruction algorithm for food volume estimation. More recently, Gao et al. [24] presented a monocular Simultaneous Localization and Mapping (SLAM) algorithm for food object reconstruction. Compared with model-based methods, the stereo vision-based methods require less human efforts during volume estimation. However, these methods are challenged by multiple tasks such as camera calibration, feature extraction, image registration, pose estimation, etc., making its implementation technically difficult. In addition, as mentioned previously, the 3D reconstruction approach requires multiple images of the same food in different views. As a result, the camera must be moved around the food while acquiring images, a difficult maneuver for a wearable camera. Although installing two cameras within a single wearable device can theoretically acquire stereo images, both the device cost and power consumption increase. Moreover, since the wearable device is small, the distance between the two cameras may be too short to produce a sufficient stereo effect for volumetric measurement.

In recent years, with the rise of deep learning technology, many computer vision tasks (e.g., image classification [25], image segmentation [26], and depth estimation [19]) have advanced significantly. As a result, there is a recent trend of applying deep learning to food volume estimation. For example, Meyers et al. [21] estimated a depth map from the 2D food image with deep Convolutional Neural Networks (CNN) and then converted the depth map to a 3D voxel representation. Considering that the estimated depth maps from RGB images may contain large errors, Lo et al. [18] instead adopted a RGBD camera to simultaneously capture color and depth images for food volume estimation. The food reconstruction is implemented by performing the Iterative Closest Point (ICP) algorithm over the front-view depth map and an inferred back-view depth map. Ferdinan et al. [27] and Lu et al. [28] took a different approach. They formulated the volume estimation as a volume regression problem from implicit 3D features transformed from the depth information.

Despite the significant progress achieved in recent years on image-based food volume estimation, most of these methods suffer from at least one of the following drawbacks: (1) requiring considerable human effort in selecting and manipulating 3D food shape models; (2) requiring a special RGBD camera, more than one RGB cameras, or moving a single RGB camera around the food during image acquisition; and (3) relying heavily on estimated depth information which is often sparsely available from images with a single-view or limited views, resulting in a large error and/or instability in estimation results.

## 3. Methodology

### 3.1. Motivation

**Figure 1.**
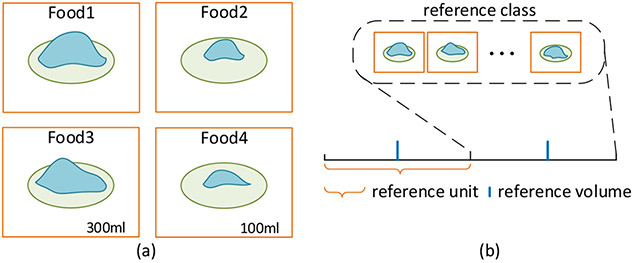
(**a**) Motivation of our method: A human can easily tell that Food No. 1 is larger than Food No. 2 if the plates in the two images have the same size. Moreover, we can roughly estimate the volume of Food No. 1 or 2 if some reference volumes (Foods No. 3 and 4) are given. (**b**) Definitions of terms: The reference class is an abstract food class that have similar volumes, the reference volume is the center volume of a reference class, and the reference unit is the interval between two neighboring reference volumes.

Instead of acquiring multiple images and reconstructing depth explicitly, we use only a single-view image to estimate food volume. Our approach to this difficult problem is to mimic human thinking in volume estimation using AI technology. Over the years, dietitians have used an intuitive method comparing the food (either in the physical world or in an image) of an unknown size with a number of sizes of common objects, such as a thumb tip, a golf ball, a deck of cards, and a baseball. The sizes of the objects close to the size of the food are mentally extrapolated to produce an estimate. This method, although not very accurate, is proven to be highly effective. Studies in psychology provide an explanation of the effectiveness in terms of the Stroop effect where the size difference of two familiar objects in an image can be rapidly perceived when their sizes are congruent with those of the real world [29]. In addition, over the process of evolution, a human becomes highly capable of not only selecting a particular object (e.g., a larger one) among the same type of objects of different sizes, but also estimating the size of one type of object in reference to another object of a different type with a known size. This is illustrated in Figure 1a where we can easily tell that Food No. 1 appears larger than Food No. 2, assuming that the plates in the two images have the same size. We can also roughly estimate the volume of any one food in the pair if the actual volume of the other food is known, provided that the plates are of the same size (bottom row). The estimation is facilitated even further if more than one reference foods of known volumes are available (Figure 1b), assuming that all plate sizes are identical.

**Figure 2.**
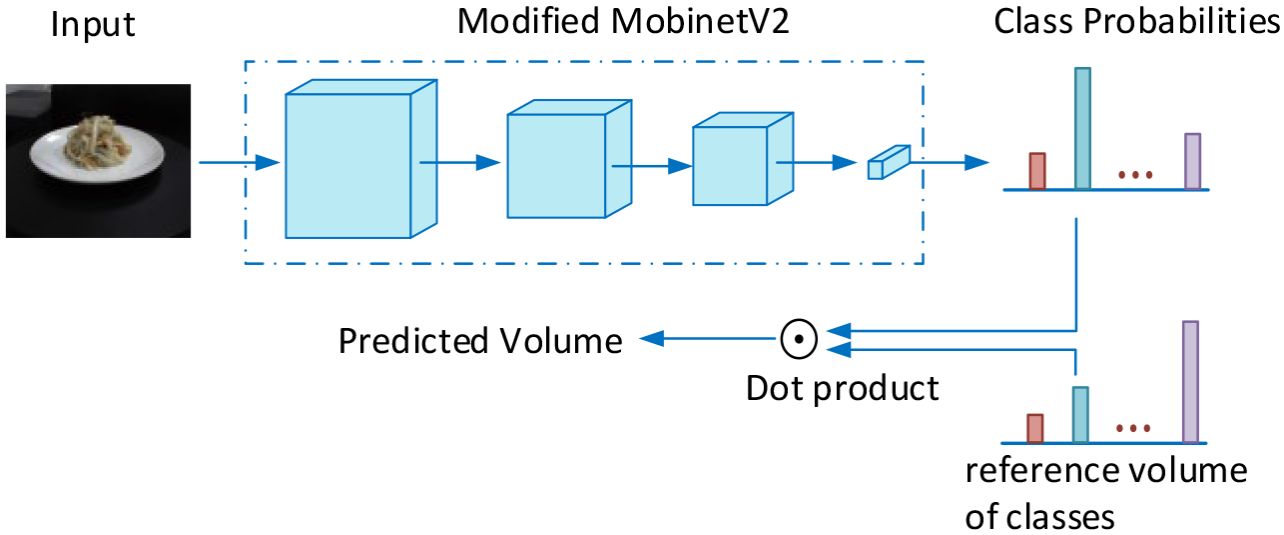
Overview of the proposed food volume estimation system which contains two stages. In the first stage, an image classification network outputs a vector of the probability values with respect to a pre-selected set of reference classes, where the classification network is modified (detail modification is shown in Table 1) from the MobileNetV2 model provided in Pytorch. In the second stage, the food volume is estimated by an inner product between the probability vector and a volume vector consisting of the volumes of reference classes.

These observations inspired us to adopt a human-mimetic strategy for food volume estimation from a single-view image using an AI system. This new strategy, shown in Figure 2, consists of two steps. In the first step, our AI system roughly classifies which reference volume (the value is known) that the observed food matches the best, just as a dietitian does in portion size estimation. In the second step, our system provides a fine-tuned volumetric estimation by comparing with multiple volumetric references and extrapolating the result, in the same process as that illustrated in Figure 1.

If we treat food images with similar volumes as an abstract class, the first step mentioned above can be interpreted as finding the closest volume class for the input image, which can be formulated as an image classification problem. For the sake of clarity, we call the volume used for class division as the reference volume and the abstract class associated with it as the reference class (see Figure 1b). Since deep CNNs such as the ResNet [30] and DenseNet [31] have shown great success in image classification, we choose from these deep learning architectures for reference class classification. In particular, we set multiple reference volumes (e.g., 200 mL, 300 mL, 400 mL) for reference class division. Each reference class is associated with numerous training food images whose volumes lie within a small range (e.g., ±50 mL) with respect to its reference volume. Thus, if a food dataset has the maximum volume of 1000 mL, for instance, we can formulate the food volume estimation as a 10-class classification problem if 100 mL is adopted as the unit of reference.

### 3.2. Neural Network for Food Classification

In order to deploy our AI technology to an embedded system within the wearable device for real-world food volume estimation in the future, we adopt the light-weight inverted residual block as our basic unit to construct a real-time food volume classification network. We first briefly review the concept of the inverted residual block and then detail our network architecture.

#### 3.2.1. Inverted Residual Block

The inverted residual block [32] was developed from the original residual block proposed in the ResNet [30]. Similarly, it is a stack of a 1 × 1 convolution layer, a 3 × 3 convolution layer, and a 1 × 1 convolution layer. The inverted residual block uses the first 1 × 1 convolution layer to expand the feature dimension (contrary to the original residual block which reduces the dimension), and the second 1 × 1 convolution layer to restore the feature dimension. In addition, the inverted residual block replaces the standard 3 × 3 convolution with a depth-wise separable (dws) convolution. This type of convolution [33] factorizes the standard convolution into a 3 × 3 depth-wise convolution and a 1 × 1 pointwise (pw) convolution, which reduces the computational cost significantly.

#### 3.2.2. Food Volume Classification Network

Based on the inverted residual block, we construct a food volume classification network, which mimics the mental process of the dietitian to determine the reference object that best matches the food volumetrically, composed of five blocks with a different output resolution. This network outputs the probabilities of the reference classes to which the food in the input image belongs. The detail of our network architecture is shown in Table 1. Block 1 consists of a 3 × 3 standard convolution layer and an inverted residual layer. Blocks 2 through 5 are stacked by different numbers of residual layers. In addition, Block 5 contains an additional 1 × 1 convolution layer to transform the feature to a higher dimension of 1024. The global pooling strategy is used to convert the feature maps to a feature vector for image classification.

For classification with respect to reference classes, we use hard labels, i.e., one-hot encoding, to supervise network training. Specifically, each training image is associated with a binary label vector that contains only one element equals to 1. The index of “1” indicates the reference class closest to the training image. However, our goal is volume estimation rather than volume size classification. A simple way to achieve this goal is to use the closest reference volume as the estimated volume. However, this could result in a significant information loss. For example, given a test image, its distances to the closest reference class and the second closest class could be very similar. Thus, we use soft predictions instead, i.e., the probabilities of reference classes that a food in the input image belongs to, to perform volume estimation. The soft predictions not only tell the closest reference class, but also give information about the relative closeness to other reference classes. This process again mimics the mental process of the dietitian who uses degrees of likeliness among a set of known objects to arrive at an estimate. Specifically, the food volume is computed by the following inner product:

(1) $$\hat{V}=\sum_{i=1}^{N}p(i)V(i)$$

where *V*(*i*) is the reference volume of class *i*, and *p*(*i*) is the probability of the *i*th reference class that the food belongs to.

### 3.3. References Volume Normalization

**Figure 3.**
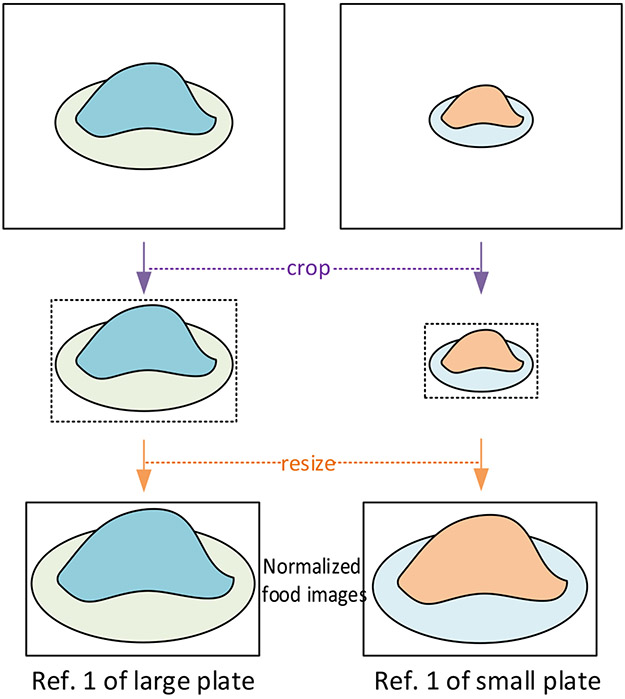
Concept of normalized references: Different food volumes can be normalized to the same or a similar reference volume by first cropping the foods from the input image and then resizing the foods to the same size.

For the AI-based volume estimation system described above, it is assumed that all the plates in the images have the same size because the plate acts as a scale reference in this case. As a result, we need to train multiple models if plate sizes are different, which degrades the generality of our method. To solve this problem, we propose to crop the food along with the plate from the images and then re-size the cropped sub-images to a fixed size (see Figure 3). Such an operation can be viewed as normalizing different sizes of plates to 1 since it enforces different plates having the same radius in pixel unit. Since the maximum food volume a plate can place is usually pre-defined (e.g., 1000 mL) according to the plate’s size, thus, the maximum reference volume within the normalized plate is also normalized to 1 (which becomes unit free due to the normalization process). After this normalization, food images with large differences in volume can have a similar normalized volume and thus can be placed in the same class. As a result, we can collect the dataset using the same plate to train the classification network and need only change the reference volumes for different plates during volume estimation. Specifically, denoting the original volume of a food as *V*, and the normalized volume as $\bar{V}$, we have:

(2) $$\bar{V}=\frac{V-V_{min}}{V_{max}-V_{min}}$$

where *V*_*max*_ and *V*_*min*_ are the maximum and minimum reference volumes, respectively. Thus, the normalized volume lies in a closed interval of [0, 1]. If we use *N* references classes for volume estimation, each reference class will cover a volume range of 1/*N*. Then, the normalized reference volume of the *i*th class equals to *i/N* − 1/2*N*. According to (1), the normalized volume estimation can be computed by:

(3) $$\bar{V}=\sum_{i=1}^{N}p(i)(\frac{i}{N}-\frac{1}{2N}).$$

Finally, according to (2) and (3), the estimated volume can be obtained by:

(4) $$\hat{V}=V_{min}+(V_{max}-V_{min})\sum_{i=1}^{N}p(i)(\frac{i}{N}-\frac{1}{2N}).$$

Thus, once the model has been trained, only the maximum and minimum reference volumes are required for de-normalization, i.e., for estimating the actual volumes in unseen plates. Next, we describe how to obtain these two values.

**Figure 4.**
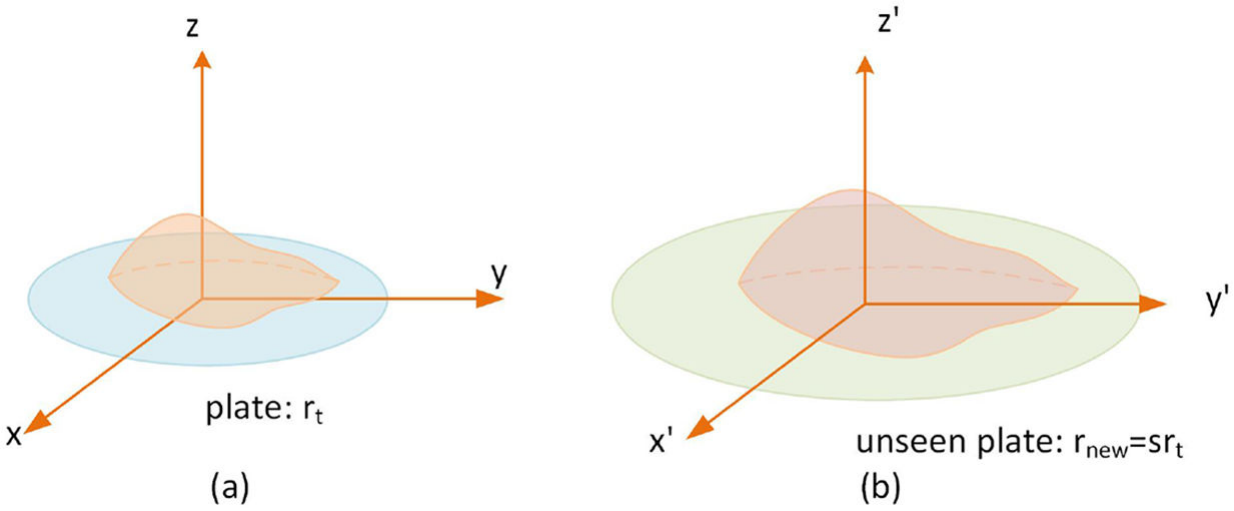
(**a**) Supposed 3D models for reference volume computation: (**b**) Scaled 3D food models.

Without loss of generality, supposing that the *n*th reference volume of the training set is obtained from a 3D model with irregular surface shown in Figure 4a, Then, the reference volume of class *n* can be computed by a triple integral defined over the 3D model:

(5) $$V_{ref\_n}^{r_{t}}=\underset{\omega }{∭}dxdydz$$

where *r*_*t*_ is the plate radius in the training images and *ω* is the region enclosed by the 3D model. Then, for an unseen (with respect to the training set) plate *r_new_* = *sr_t_*, the 3D model for *n*th reference volume computation can be obtained by scaling the model used in a training set with a factor *s* in all three dimensions (Figure 4b), thus, the *n*th reference volume of plate *r*_*new*_ can be computed by:

(6) $$V_{ref\_n}^{r_{new}}=∭d(sx)d(sy)d(sz)=s^{3}V_{ref\_n}^{t}.$$

Thus, given food images placed in an unseen plate, to obtain the maximum and minimum reference volume for volume estimation, we only need the plate radius to compute *s*.

## 4. Datasets

It is well known that the deep network requires a large amount of annotated data for training. However, most current food datasets (e.g., PFID [34], UECFOOD-100 [35], Food-101 [36]) are designed for food recognition, rather than volume estimation since these datasets has no information about food volumes. For this reason, most existing deep learning-based studies on food volume used self-collected datasets for training. For example, Li et al. [22] collected a fruit dataset to train their fruit volume estimation network. Similarly, Meyer et al. [21] collected a RGBD food dataset for their depth-based food volume estimation network using the Intel RealSense camera. Unfortunately, these datasets cannot be utilized to train our AI system because they are not publicly available, highly specialized for certain foods (e.g., fruits), or focused on depth. Since it is extremely time-consuming to measure a large number of foods as volumetric truths for neural network training, we first generated two virtual food datasets using computer simulation to validate our human-mimetic method quickly. After the effectiveness our method was proven, we then applied our method to real-world food images. This two-step approach allowed us to circumvent the volume truth measurement problem initially and accelerated the design of our AI system.

### 4.1. Virtual Food Dataset

**Figure 5.**
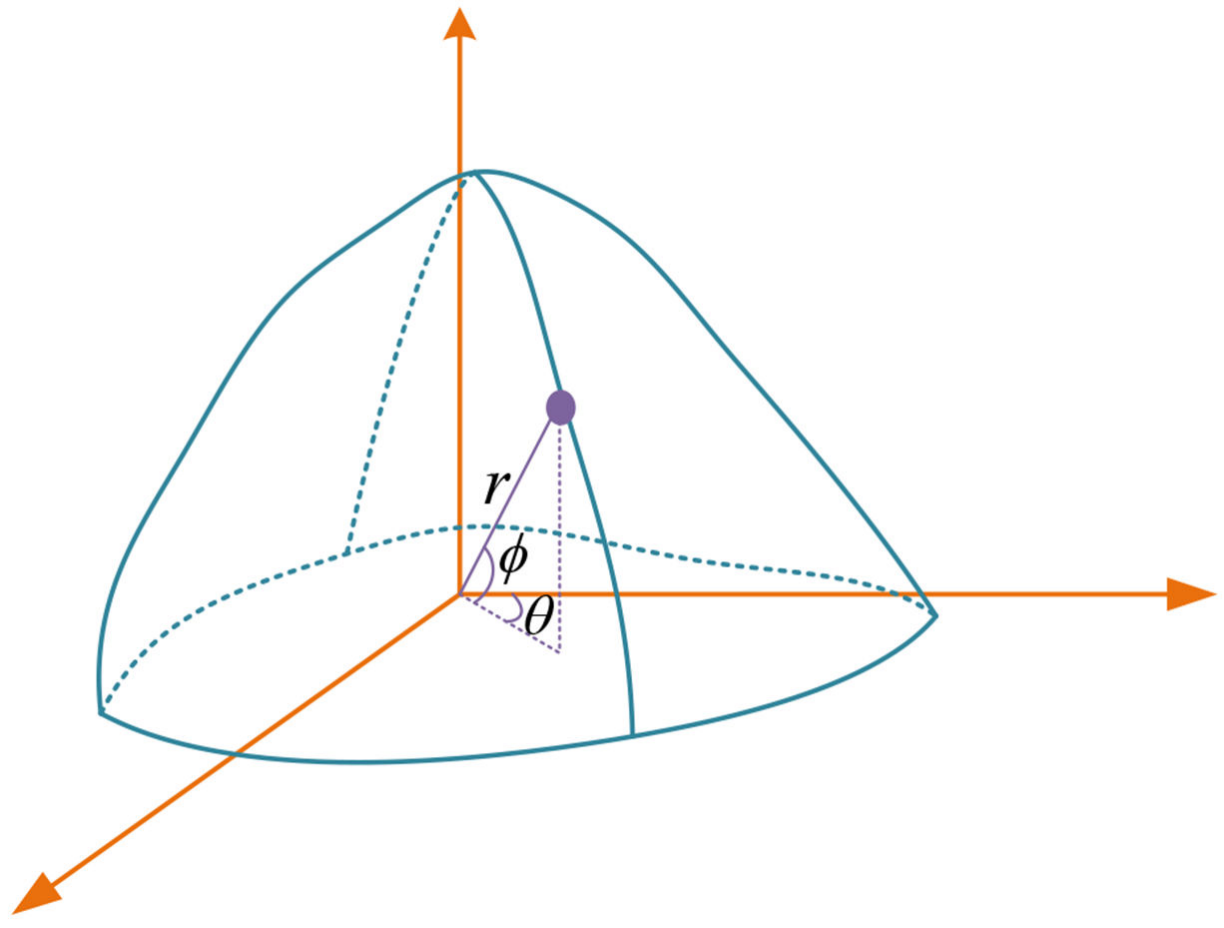
Spherical coordinates system for VFD generation.

**Figure 6.**
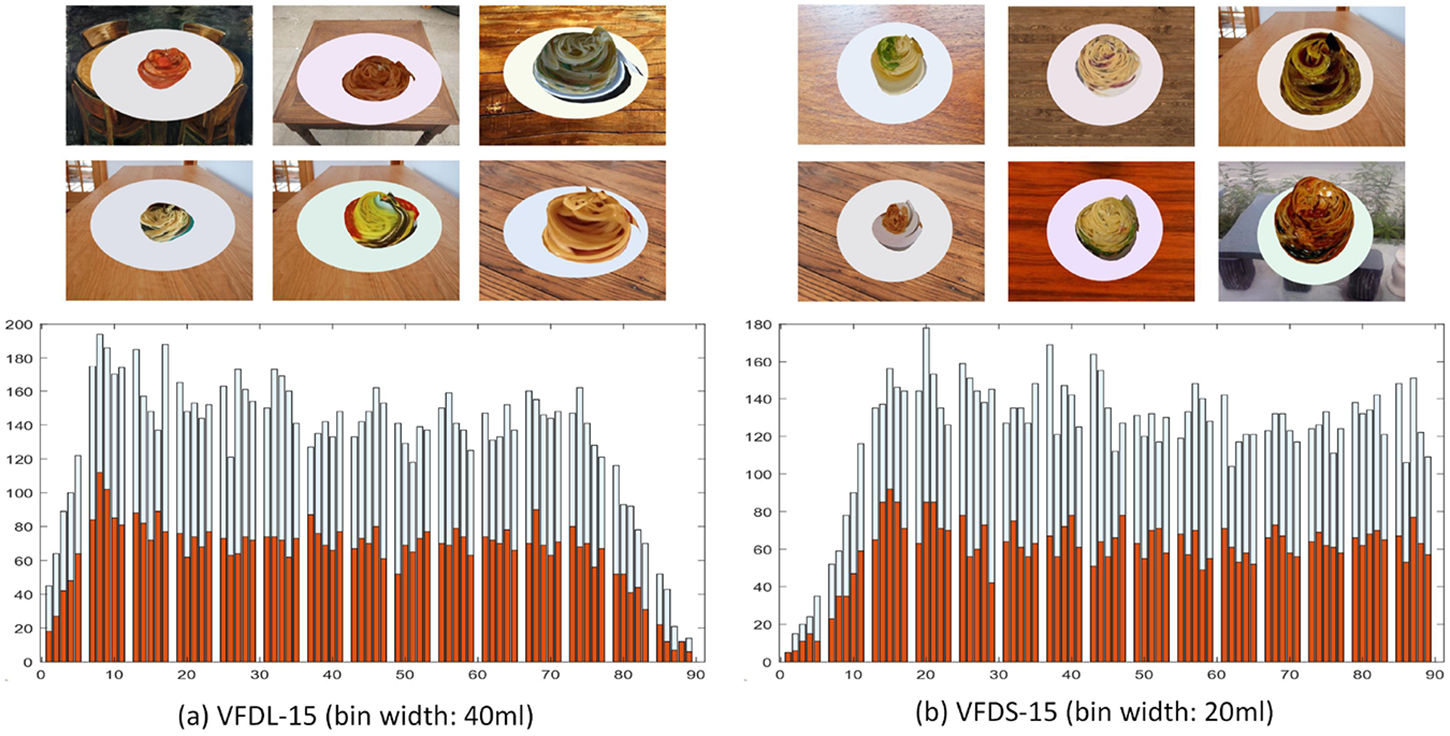
(**a**) Examples and histograms of VFDL-15; (**b**) Examples and histograms of VFDS-15. The blue and orange bins are histograms of a training set and test set, respectively. Note that the plate size of VFDL-15 is different from the one of VFDS-15 although they look similar.

The key problem of producing a Virtual Food Dataset (VFD) is to simulate complex and varying food shapes with a computational algorithm. In order to obtain a sufficient variability in a simulation result, we defined a spherical coordinate system shown in Figure 5 and generated a cloud of 3D points (*r, θ, ϕ*). The azimuthal angle *θ* and polar angle *ϕ* incremented equally in ranges of 0–360° and 0–90°, respectively. In contrast, the radial distance *r* was generated randomly with a Gaussian distribution of $N(\hat{r},\sigma )$, where $\hat{r}$ and *σ* are the mean and standard deviation of this distribution. Once the point cloud was generated, we applied a low-pass filter, implemented by a 2D mask across overlapped (*θ, ϕ*) patches. The low-pass filtering produced a smooth surface of a random shape. We adjusted the coefficients of the mask and the parameters of the distribution ($\hat{r}$ and *σ*) experimentally by observing the simulation results. In order to improve the appearance of virtual foods, we wrapped each generated surface with randomly selected real food images. The results of our computer simulation are exemplified in the top two rows of Figure 6. The background images and food images used for wrapping are downloaded from the Internet. In these virtual images, the food occupied a large part of each image frame to simulate the cropping effect (described in Section 3.3).

In order to evaluate our AI method in handling foods with different volumetric ranges, two virtual datasets were generated with different minimum and maximum volumes. In the first dataset, 15 classes were utilized, and the minimum and maximum volumes were 400 mL and 3400 mL, respectively. In the second dataset, 15 classes were utilized again but the volumetric range was smaller, between 200 mL and 1700 mL. Note that the minimum volumes of the two VFDS were not zero because random processes that we utilize tend to produce fewer samples when the volume was close to zero. For convenience, we called these two datasets VFDL-15 and VFDS-15, respectively (“L” and “S” represents large and small, respectively). Finally, we divided each dataset into a training set and a test set with a roughly 2:1 ratio (10,003:4889 for VFDL-15 and 9205:4489 for VFDS-15).

The bottom row in Figure 6 shows the distributions of the two VFDs constructed by us. We have made our VFD publicly available at https://drive.google.com/file/d/1CobbDAw_QeZfitBPleZGBnXY0nkntKtw/view?usp=sharing (accessed on 25 June 2021).

### 4.2. Real Food Dataset

We first established a Real Food Dataset (RFD) consisting of 1500 images captured by a stationary camera. This RFD contained 50 Chinese foods of a university cafeteria in China. In addition, for each food, multiple images were taken by turning around the table where the food placed, providing an ideal dataset for training and testing our AI-based volume estimation system.

**Figure 7.**
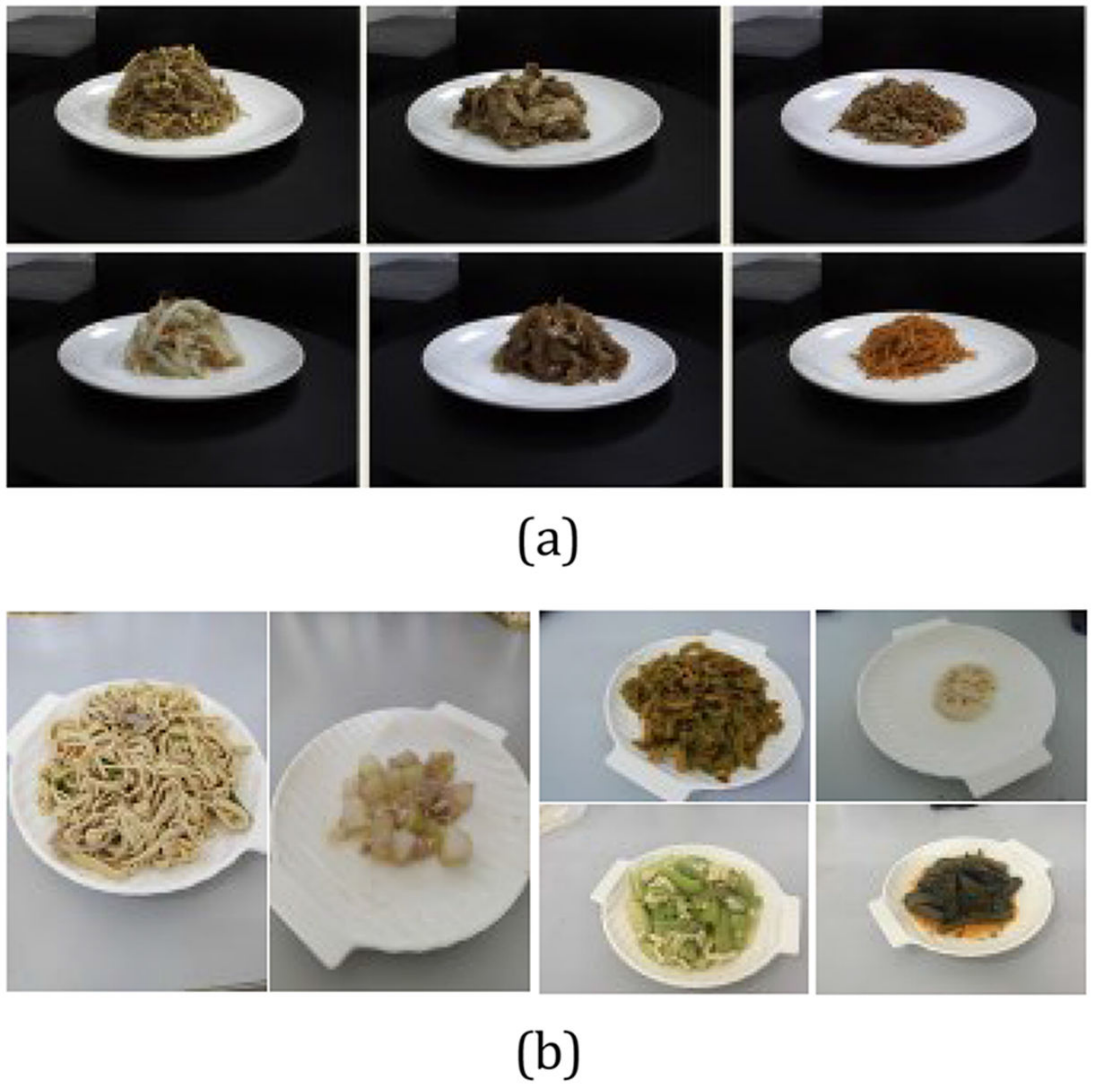
(**a**) Examples of IRFD, (**b**) Examples of GRFD. The images in IRFD are captured by a stationary camera while the images of GRFD are captured by smartphones.

In order to evaluate the performance of our system for real life cases where a single-view image is taken at an unrestricted view angle, we established another RFD using personal mobile phones (brands unrestricted) to capture food images. This dataset consisted of 416 images. Unlike the previously case, these images were taken with user-determined view angles although views take directly above the food were discouraged. For convenience, we call this dataset a general RFD (GRFD). Likewise, the previous dataset is called an ideal RFD (IRFD). The volume of IRFD ranges from 110 to 410 mL, and the volume of GRFD ranges from 66 to 630 mL. As shown in Figure 7, images in GRFD have considerable differences in view angles than the images in IRFD.

## 5. Experiments

### 5.1. Experimental Setups

#### 5.1.1. Training Policy

We trained our deep neural network using the standard Stochastic Gradient Descent (SGD) algorithm. The batch size was set to 128 × 224 × 224. We set the learning rate to 0.01 and divided it by 10 after every 5000 steps. The total training steps were set to 15 K and 5 K for VFD and RFD, respectively.

#### 5.1.2. Data Augmentation

Since our food volume estimation system needs to see the entire 2D food, we employed only “random mirror” for data augmentation.

#### 5.1.3. Evaluation Protocol

We computed top1 and top3 classification error to evaluate the classification accuracy, where “top” refers to the classes that are closest to the truth class. For example, for volume class 3, the three classes closest to it are the class 2, class 4, and 3 itself. Thus, for class 3, the top3 accuracy computes the ratio of samples that are classified into class 2, 3, and 4 to the total samples. More formally, the top1 and top3 classification error can be computed by (7) and (8) respectively.

(7) $$top1=\frac{\sum_{i}^{N}\delta ({\hat{l}}_{i}l_{i})}{N}$$

(8) $$top3=\frac{\sum_{i}^{N}\left(\delta ({\hat{l}}_{i},l_{i}-1)\;\|\;\delta ({\hat{l}}_{i},l_{i})\;\|\;\delta ({\hat{l}}_{i},l_{i}+1)\right)}{N}$$

where ${\hat{l}}_{i}$ and *l_i_* are the predicted volume class and truth volume class of sample *i*, respectively *δ*(*x, y*) is an indicator function written by:

(9) δ(x,y)={1ifx=y0otherwise.}

We also computed the Mean Relative Volumetric Error (mRVE) as a measure of accuracy for volume estimation. For each estimation, the RVE was computed by:

(10) $$RVE=\frac{|V_{p}-V_{t}|}{V_{t}}$$

where the *V_p_* and *V_t_* are the computed and measured volumes, respectively. The mRVE is then obtained by averaging the RVE value of all test samples.

#### 5.1.4. Computing Systems

All the experiments were conducted on a computer equipped with the Intel Xeon E5-1630 (8 cores, 3.7 GHz) CPU, Titan X (12G) GPU, and 32G RAM. We trained our AI system using the Tensorflow software platform [37].

### 5.2. Experiments on VFD

#### 5.2.1. 15 Reference Classes

We first tested our human-mimetic method using the VFDL-15 and VFDS-15 datasets separately. The experimental results are shown in Table 2. The first and second rows of mRVE displays the performance of using soft predictions and hard predicted label for volume estimation, respectively. Several important observations and conclusions are described as follows.

**Figure 8.**
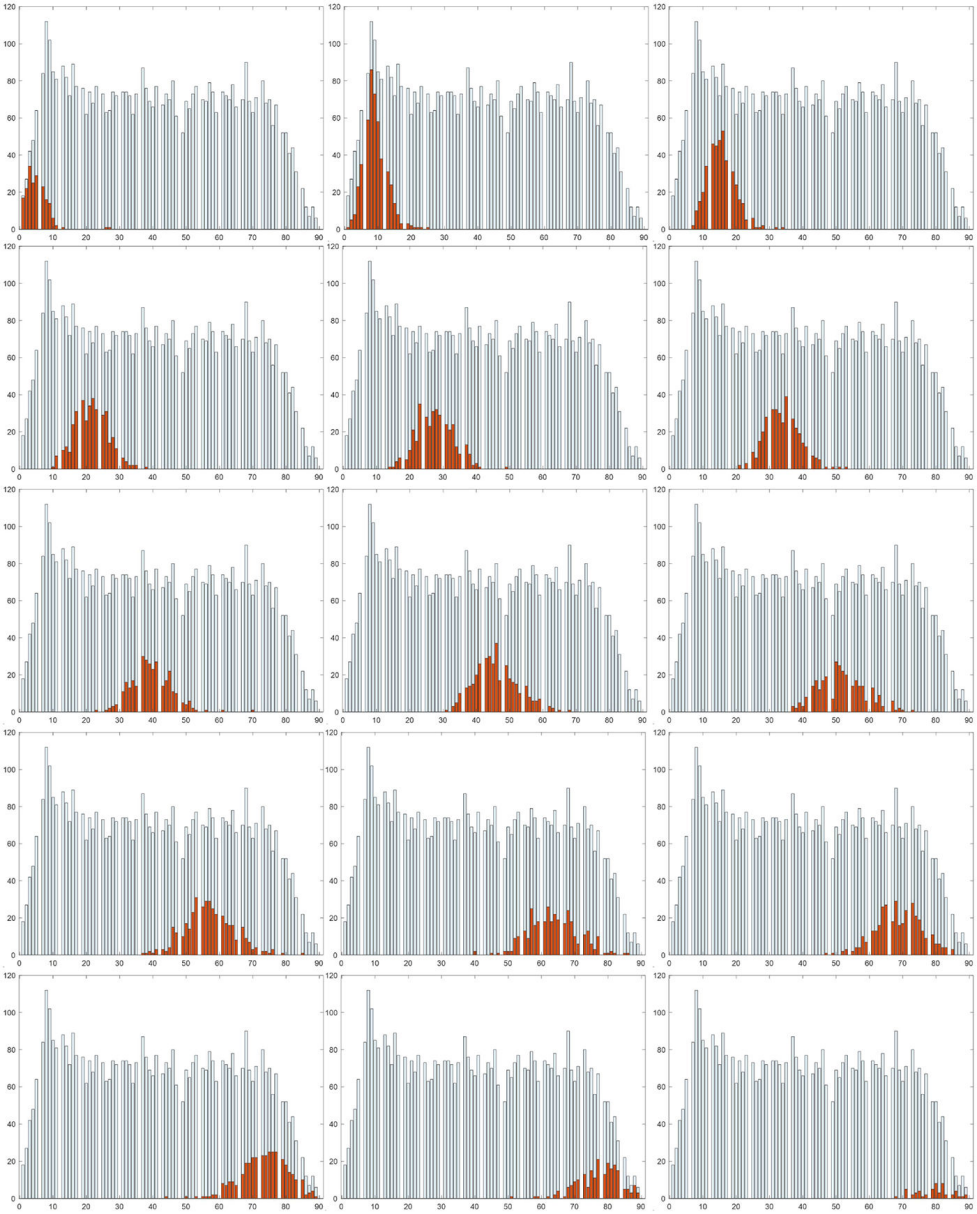
Histograms (40 mL for bin width) of classification results for VFDL-15. White distributions indicate the test set (for clarity, all classes are shown and separated by a blank bin). Orange distributions (one class for each panel) represent classification results for classes 1 through 15.

1. Our human-mimetic AI system achieved a 86.7% and 83.7% top3 accuracy on VFDL-15 and VFDS-15, respectively, which demonstrated that this system is able to find the three closest volume reference classes of the input, in a similar way that a human uses to compare a food size with the sizes of a set of reference objects. In addition, according to the histograms shown in Figure 8, it is unlikely for our method to classify the input images to the reference classes far away from their true reference class. Thus, although the top1 accuracy was relatively low, our method still achieved 8.7% and 8.7% mRVE on VFDL-15 and VFDS-15, respectively.
2. Most top1 classification accuracies achieved on large volume classes were fewer than 40%, suggesting that the food volume classification model cannot distinguish large volume classes very well. This was mainly because the relative volumetric changes between large volume classes were much smaller than the changes across small classes, which made large volume classes less differentiable. Nevertheless, the volume estimation errors in large volume classes were typically smaller than the ones of small volume classes. This is reasonable since the RVE is more sensitive to the absolute error at small classes according to (10). For example, given an absolute error equal to 100, for the VFDL-15 dataset, the mRVE of class 1 (400–600 mL) is in the range of 16.7% to 25%, while the mRVE of class 15 (3200–3400 mL) lies between 2.9% to 3.1%.
3. Using soft predictions for volume estimation achieved lower mRVE than using hard predictions on both VFDL-15 and VFDS-15, proving the effectiveness of our soft predictions-based volume estimation mentioned in Section 3.2.2.
4. Not surprisingly, our method achieved a better performance on a VFDL-15 dataset for the reference classification task according to the top1 and top3 accuracy measures. However, for the volume estimation task, our method showed a similar mRVE on the VFDS-15 and VFDL-15. Together with observation 2, it can be concluded that a better reference classes classification accuracy, which usually requires a larger interval between neighbor classes, does not imply a better volume estimation result.
5. We achieved 8.7% mRVE on the VFDS-15 dataset that has a similar volume range to that in the real world, which demonstrated strongly the effectiveness of our human-mimetic approach.

#### 5.2.2. Increased 30 References Classes

According to Observation 3 mentioned above, a better accuracy in reference class classification does not imply a better volume estimation. While fewer reference classes usually result in a better classification accuracy, our goal is volume estimation rather than classification. Therefore, we further increased the number of reference classes and studied the resulting volume estimation performances. In particular, we used 30 reference classes for the original VFDL-15 and VFDS-15, forming VFDL-30 and VFDS-30 in the new experiment. The volume intervals between neighboring reference classes were chosen as 100 mL and 50 mL for VFDL-30 and VFDS-30, respectively. Our experimental results are shown in Table 3.

As expected, both top1 and top3 classification accuracies decreased significantly when the number of reference classes was increased from 15 to 30 for both VFDL and VFDS. Since each class of VFD-15 was split into two classes in VFD-30, the top1 and top3 errors for classes in VFD-15 should correspond to top2 and top6 errors for classes in VFD-30, respectively. In this context, the top1/top3 accuracy of VFDL-30 and VFDS-30 is 40.8%/80.9% and 38.0%/77.3%, respectively. In addition, the number of training samples of each class in VFD-30 was decreased significantly compared with those in the VFD-15. In other words, a better classification accuracy is expected for VFD-30 if more samples are available.

#### 5.2.3. Bias Analysis

**Figure 9.**
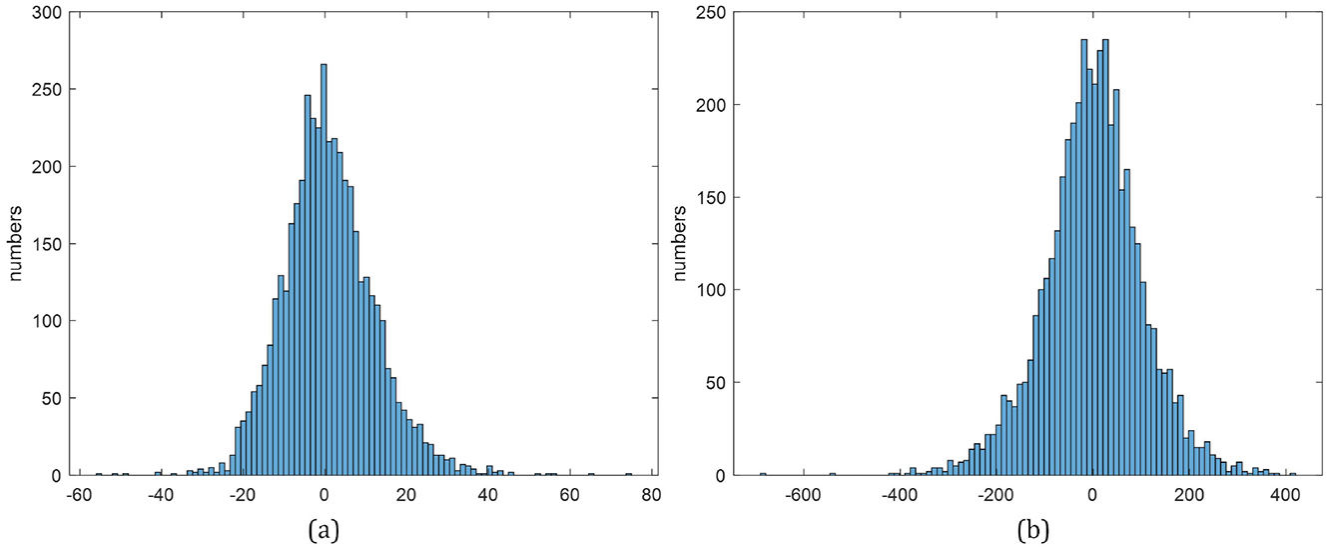
(**a**) Histograms of relative errors (%) on VFDS-30, (**b**) Histograms of absolute errors (mL) on VFDS-30.

In order to check whether our human-mimetic AI system produced biased volumetric estimates, we investigated the distribution of volumetric errors shown in Figure 9. It appears that the error distribution, after a normalization, can be approximated by a Gaussian distribution centered at zero, suggesting that our AI-based volume estimator is unbiased.

#### 5.2.4. Training with Normalized Reference Class

In order to demonstrate the effectiveness of our normalization approach described in Section 3.3, we mixed the training data of the VFDL-15 and VFDS-15 and obtained a combined training set consisting of 19,208 images. Then, we adjusted the reference volume for each dataset during the test stage according to (4). The experimental results are shown in Table 4. By comparing Table 2 with Table 4, it can be observed that training with mixed datasets achieved a better performance than training with each dataset individually. In particular, top1 accuracy, top3 accuracy, and mRVE on the VFDL-15 dataset were improved by 1.7, 1.4, and 0.2 percentage points, respectively, these three quantities were improved by 0.6, 0.9, and 0.2 percentage points, respectively, for the VFDS-15. These improvements may have resulted from the increase in the number of training samples for each class.

Our experiments demonstrated that food images can be placed in the same class as long as they share similar normalized volumes, regardless of their actual volumes, as we stated previously in Section 3.3.

### 5.3. Experiments on RFD

We first tested our human-mimetic method using the IRFD dataset. Similar to the VFD results, we also experimented two reference units to divide the reference classes. When 100 mL was adopted as the reference unit, the available food images could only be divided into 3 classes. Thus, we did not list the top3 accuracy since they were 100% in this case. It can be observed from Table 5 that our AI system produced similar mRVEs when using different reference units. In addition, our AI system produced an mRVE less than 15% for most individual classes and 11.6% overall, which are satisfactory results in food volume estimation from single-view images.

Next, we applied our method to the GRFD dataset which contained 416 images with measured volumes. We divided these images into the training (242 images) and testing (174 images) set. Since the GRFD had significantly fewer training samples (960 for IRFD vs. 242 for GRFD), and the view angles of the images in GRFD are more varied than those in IRFD, the estimation error was larger. Nevertheless, a reasonable performance was achieved with a 20.1% mRVE (Table 6).

Since the images were randomly divided into the training and test sets in previous experiments, the same type of food could be found in both the training and test sets. To investigate the capability of our human-mimetic system in handling unseen foods, we performed an experiment where the images of IRFD were divided into the training or test sets with different food types. In other words, all food types in the test set cannot be found in the training set. Note that we did not conduct this experiment on the GRFD dataset because a large number of training samples would be required to enable the deep network to handle unseen foods. With the same consideration, we used three classes rather than five classes. We finally obtained 960 training images and 540 test images. It can be observed from Table 7 that our AI system produced 12.5% mRVE even with all the test foods being new to the network. This experiment demonstrated the our human-mimetic volume estimation system was able to focus its “mental activity” on the food volume, rather than other food features.

### 5.4. Comparison with Other Methods

In this section, we compared our method with a number of existing methods. A general criterion is to choose the state-of-the-art methods that report performance on the same dataset for a fair comparison. Unfortunately, there has not been a well-recognized dataset for food volume estimation according to a recent published comprehensive review [38]. Instead, most methods reported performance on their own collected data that usually is not publicly available. Thus, for comparison purposes, we chose some best-known food volume estimation methods that used evaluation criteria, i.e., relative error, similar to ours and that were published after 2015. Note that some negative relative errors in Table 8 can be easily transformed into our evaluation criteria by taking the absolute value.

As shown in Table 8, we compare our method with these methods both quantitatively and qualitatively. Compared with eye-measurement, which inspired our method initially, our method achieved similar or better performance, proving the effectiveness of our human-mimetic volume estimation. When compared with other automatic volume estimation methods, our method showed an uncompetitive performance in accuracy. Nevertheless, these methods typically required more information (e.g., distance to camera, multi-view images, sizes of checkerboard) than our method, making them difficult to implement with wearable devices with limited hardware resources. For example, the VD meter [42], a specialized laboratory instrument for food volume estimation, achieved the best performance on mRVE (0.83–5.23%), but it required 192 2D images in different views, which were obtained from an array of cameras mounted on a curved, stationary arm, to implement the 3D food reconstruction. Note that the images of our IRFD dataset were also obtained from one of the cameras of the VD meter. Similarly, Hassannejad et al. [13] also used multi-view images and reported relative errors ranging from 1.7–19.1%. However, it is difficult for an unconscious wearable camera to obtain these multi-view images. Among the methods based on depth information, Lo et al. [18] achieved the relative errors ranging from 3.3–9.4%, which outperformed our method by an average of 10 percentage point if not considering the difference between the database used. However, these methods typically rely on special depth cameras to obtain the depth image. In contrast, our method only needs a single RGB image for volume estimation. In addition, many of these compared methods used only a small number of food objects to test their performance. For example, the MuseFood [39] used only three food items, i.e., three food images, to evaluate its volume estimation accuracy. Point2Volume [41] adopted more food objects but the number is still limited to 11. In contrast, we adopted more than 174 food images to evaluate our food volume estimation algorithm.

Thus, the most significant advantage of our method is that we relax the requirements for scale reference and 3D information, making it more suited for dietary assessment based on wearable devices. As stated previously, a scale reference such as a checkerboard must be carried by the individual and placed near the food. This is an unwelcome procedure that is usually difficult to implement. On the other hand, acquiring additional 3D information would either increase human effort or the size of wearable devices.

It should be noted that our method has one major limitation in that we assume that the food plate can be accurately detected from real-world images, which usually contain irrelevant backgrounds such as a table, wall, and people. This assumption is made according to the recent success in object detection achieved by deep learning. In other words, our method relies on a high-quality object detection algorithm to crop the food plate firstly from real-world images.

## 6. Discussion

In this section, we discuss several important issues related to the automatic approach to food volume estimation.

### Relative Food Volume:

As mentioned previously, the most stringent requirement in image-based volume estimation is to provide a scale reference (or a fiducial marker) in the image, such as a checkerboard card. Although a person can place this reference within the view of the camera, it is inconvenient in practice. Since, in many parts of the world, foods are usually placed in a plate (mostly a circular shape) for serving, using the plate as a scale reference is a more suitable choice. For a circular plate, in particular, only its diameter needs to be known. However, this parameter still requires human effort for measurement. As a result, billions of food images existing on the websites cannot be utilized for volume estimation because the plate diameter is unknown, which is a great waste of resources. In this work, we presented a normalization method in Section 3.3 where a food image is cropped and normalized. As a result, the plate, regardless of its actual size, becomes standardized and unitless. For such a normalized image, our AI system is able to estimate the relative volume for food images without information about the plate size. The relative volume can be later converted easily to the true volume when the plate diameter becomes available.

### Multiple Foods in One Plate:

Most experiments in this paper were designed for evaluating the effectiveness of the AI approach to food volume estimation. For simplicity, we limited it to the case where each plate contains only one type of food. In practice, however, more than one foods are occasionally placed in a single plate. Although automatic separation of multiple foods is beyond the focus of this paper, we briefly discuss the computational procedure called image segmentation. Decades of research in this field have produced a rich set of algorithms to label and separate objects. Since food objects have complex shapes and textures, the traditional algorithms have limited success. Recently, deep learning-based semantic segmentation algorithms [43-45] have emerged. Using these algorithms, different foods can be first recognized and separated by a deep neural network [18,21]. Then, the human-mimetic method presented in this paper can be applied to each food for volume estimation.

### AI Perspective:

Although using AI for a dietary assessment is still in its initial infancy, we believe that this approach has a great potential to advance nutrition science and dietetics significantly. As the research on this approach progresses, it becomes increasingly clear that at least some, and perhaps the entire, previously time-consuming self-reporting tasks can be passed to a robotic system which is unbiased, objective, and highly accurate. If successful, this new approach will exert a strong impact on public health in producing quantitative, unbiased dietary data for preventing and controlling diet-rated chronic diseases.

### Deployment to Wearable Devices:

The food volume estimation method proposed in this paper is deployed to the ebutton, a wearable device designed by our team, for objective dietary assessments. The eButton has a 2 GB RAM, an 8 GB NAND flash memory, and a microprocessor with the 32-bit ARM Cortex A9 architecture and four processing cores clocked at a maximum of 1.4 GHz [46]. It is also equipped with a wide-angle camera to capture ego-centric images. For our deep learning-based food volume estimation model, its computational load is about 4.7 G FLOPs given images in 360 × 720 resolution. As the FLOPs performance of our eButton is about 44.8 G FLOPs, the eButton has enough computational resource for deploying our food volume estimation model if not considering real-time requirements. However, our method cannot yet achieve real-time performance on the embedded CPU system. Thus, we currently did not deploy the proposed method to the eButton and instead perform off-line image analysis for dietary assessment. Nevertheless, we believe that our work will help future study on online dietary assessments with the development of hardware.

## 7. Conclusions and Future Works

In this paper, we presented an image-based automatic method for food volume estimation, aimed towards solving a long-standing problem in nutrition science where dietary assessment is subjective and time-consuming. We took advantage of recently developed AI technology and developed a human-mimetic system that imitates a dietitian’s mental process by comparing the food size with the sizes of commonly known objects. In particular, we showed that food volume estimation can be formulated as an image classification problem if we treat images with similar volumes as a reference class, which makes it possible to estimate food volume from a single RGB image without using 3D information. Moreover, we showed food images with different volumes can be also placed into the same class for network training as long as they have similar normalized volume. Then, we only need to adjust real volumes of the normalized reference classes for different plates during the testing process. Based on this approach, we pre-defined a number of references with ascending volumes acting as virtual volumetric gauges stored in a computer’s memory. Our AI system then classifies the observed food into a set of probabilities of the reference volumes. Finally, our AI system produces the best-guess volume based on the stored volumes and the computed probability vector. Our experimental results have shown that this human-mimetic approach is both accurate and robust, capable of producing a reasonable estimate from a 2D image which contains only partial 3D information. We have also developed a new normalization procedure allowing a collection of different food volumes into the same reference class, which greatly facilitates the training process for the deep neural network. In addition, we introduced the relative volume concept based on the normalization procedure for the practical cases where the plate diameter is not available. Our human-mimetic method has a potential to pass the time-consuming food portion size estimation task to an unbiased and well-trained robotic system, liberating humans from the time-consuming portion size estimation task and allowing them to improve their diet based on automatically and objectively performed dietary assessment results.

Based on our current work, future studies will be conducted from the following two aspects. First, we will build a larger food dataset to promote research in our current work. Second, as stated previously, a plate contains only one type of food in this paper, however the method of estimating volumes from a plate that contains multiple types of food using our method is also an interesting topic.

## Figures and Tables

**Table 1.** Network architecture for food volume estimation.

Stage	Output Stride	Components
block1	2	$3\times 3\;std\;conv,32\;\left[\begin{array}{c} 1\times 1\;pw\;conv,\;96\\ 3\times 3\;dws\;conv,\;96\\ 1\times 1\;pw\;conv,\;16 \end{array}\right]$
block2	4	$\left[\begin{array}{c} 1\times 1\;pw\;conv,\;192\\ 3\times 3\;dws\;conv,\;192\\ 1\times 1\;pw\;conv,\;32 \end{array}\right]\times 3$
block3	8	$\left[\begin{array}{c} 1\times 1\;pw\;conv,\;384\\ 3\times 3\;dws\;conv,\;384\\ 1\times 1\;pw\;conv,\;64 \end{array}\right]\times 3$
block4	16	$\left[\begin{array}{c} 1\times 1\;pw\;conv,\;576\\ 3\times 3\;dws\;conv,\;576\\ 1\times 1\;pw\;conv,\;96 \end{array}\right]\times 4$
block5	32	$\left[\begin{array}{c} 1\times 1\;pw\;conv,\;960\\ 3\times 3\;dws\;conv,\;960\\ 1\times 1\;pw\;conv,\;160 \end{array}\right]\times 3\;3\times 3\;std\;conv,1024$

**Table 2.** Experimental results on VFDL and VFDS with 15 classes division.

		Classes															Overall
		1	2	3	4	5	6	7	8	9	10	11	12	13	14	15	
VFDL-15	top1	63.8	67.6	56.1	46.7	41.3	44.5	35.7	39.6	30.1	36.9	28.6	28.7	35.4	36.8	16.9	42.1
	top3	100	98.3	96.8	96.1	93.4	89.6	84.3	83.2	83.3	78.0	76.7	75.5	81.2	83.2	55.9	86.7
	mRVE	15.1	11.1	10.6	9.9	9.7	9.1	9.3	8.0	7.6	7.3	7.3	6.8	5.9	5.0	8.2	8.7
		15.9	12.3	11.9	11.1	10.7	9.8	10.2	8.9	8.6	8.0	9.0	7.6	6.2	5.7	8.4	9.6
VFDS-15	top1	54.2	60.3	58.8	50.3	43.7	37.0	40.4	40.0	34.7	32.4	25.4	25.9	19.7	31.4	50.8	39.5
	top3	95.8	96.9	96.7	95.4	92.2	85.5	82.0	85.0	79.8	75.9	74.2	69.3	69.1	85.4	80.1	83.7
	mRVE	19.6	12.8	10.7	10.1	9.4	10.2	9.2	8.4	8.1	8.2	8.4	7.8	7.2	5.4	5.3	8.7
		19.8	13.7	11.4	11.1	10.7	10.8	10.3	8.7	8.6	9.0	9.2	8.7	8.0	6.2	5.1	9.4

**Table 3.** Experimental results on VFDL and VFDS with 30 class division.

	Top1	Top3	mRVE
VFDL-30	22.2	58.7	8.7
VFDS-30	21.2	55.1	8.6

**Table 4.** Experimental results with mixed training but separate tests for VFDL and VFDS.

	Top1	Top3	mRVE
VFDL-15	43.8	88.1	8.5
VFDS-15	40.1	84.6	8.5

**Table 5.** Experimental results on the IRFD dataset.

		Classes					Overall
		1	2	3	4	5	
100 mL	top1	89.0	82.4	60.7	-	-	79.6
	mRVE	13.0	10.8	11.8	-	-	11.7
50 mL	top1	78.4	27.5	79.4	35.0	75.4	67.4
	top3	97.3	100	87.6	100	88.4	93.5
	mRVE	13.2	17.7	8.9	11.6	8.9	11.6

**Table 6.** Experimental results on the GRFD dataset.

		Classes					Overall
		1	2	3	4	5	
100 mL	top1	100.0	35.0	40.0	0.0	59.6	42.5
	top3	100	100	85.0	97.6	100	96.0
	mRVE	25.8	27.3	20.9	19.1	15.3	20.1

**Table 7.** Experimental results on IRFD. The foods in the test set were unseen in the training set.

		Classes			Overall
		1	2	3	
100 mL	training samples	350	400	210	960
	test samples	210	200	130	540
	top1	88.1	76.0	78.5	-
	mRVE	13.6	14.0	8.3	12.5

**Table 8.** Comparison with other methods. n/a means not applicable, ‘-’ represents the value was not reported in the corresponding paper.

Method	Scale Reference	Input	Core Idea	Error
MuseFood [39]	Depth	RGB Image (Top + Side View)	Differential Modeling	−0.27~12.37% Test dataset: 3 food items
Eye-Measurement [39]	n/a	RGB Image	Visually Gauged by Human	−13.84~22.87% Test dataset: 3 food items
Hassannejad et al. [13]	Checkerboard	Multi-View (6) RGB Images	3D Modeling with Structure from Motion	1.70~19.10% Test dataset: 10 food items
im2calories [21]	Depth	RGB + Depth Image	3D Reconstruction with Deep Learning	- Test dataset NFood-3D dataset
Fang et al. [40]	Depth	Gray + Depth Image	3D Voxel Representation from depth	11.00~33.90% [38] Test dataset: 10 food objects
Lo et al. [18]	Depth	Depth Image (Front + Back View)	3D Reconstruction with Iterative Closest Point	3.30~9.40% Test dataset: 8 synthetic food objects
Point2Volume. [41]	Depth	RGB + Depth Image	3D Point Cloud Completion	15.32% Test dataset: 11 food items
VD Meter [42]	-	Multi-View (192) RGB Images	3D Reconstruction	0.83~5.23% Test dataset: 6 food items
Ours	Learned	Single RGB Image	Reference Volume Classification	11.60~20.10% Test dataset: 174~540 food images

## Data Availability

The datasets used and/or analyzed during the current study are available from the corresponding author on reasonable request.
